# The inferior turbinate flap in skull base reconstruction

**DOI:** 10.1186/1916-0216-42-6

**Published:** 2013-01-31

**Authors:** Jonathan Yip, Kristian I Macdonald, John Lee, Ian J Witterick, Gelareh Zadeh, Fred Gentili, Allan D Vescan

**Affiliations:** 1 Faculty of Medicine, University of Toronto, Toronto, ON, Canada; 2 Department of Otolaryngology – Head and Neck Surgery, University of Toronto, Toronto, ON, Canada; 3 Division of Neurosurgery, University of Toronto, Toronto, ON, Canada; 4 University of Toronto, Mount Sinai Hospital, 600 University Avenue, Room 401, Toronto, ON, M5G 1X5, Canada

**Keywords:** Inferior turbinate, Skull base defect, Endoscopic surgery, Reconstructive surgical procedures, Pedicled flap

## Abstract

**Background:**

As the indications for expanded endonasal approaches continue to evolve, alternative reconstructive techniques are needed to address increasingly complex surgical skull base defects. In the absence of the nasoseptal flap, we describe our experience with the posterior pedicle inferior turbinate flap (PPITF) in skull base reconstruction.

**Design:**

Case series.

**Setting:**

Academic tertiary care centre.

**Methods:**

Patients who underwent reconstruction of the skull base with the PPITF were identified. Medical records were reviewed for demographic, presentation, treatment, follow-up, surgical and outcomes data.

**Main outcome measures:**

Flap survival, adequacy of seal, and complications.

**Results:**

Two patients with residual/recurrent pituitary adenomas met the inclusion criteria. The nasoseptal flap was unavailable in each case due to a prior septectomy. Salvage of the original nasoseptal flap was not possible, as it did not provide adequate coverage of the resultant defect due to contraction from healing. All PPITFs healed uneventfully and covered the entire defect. No complications were observed in the early post-operative period. Endoscopic techniques and limitations of the PPITF are also discussed.

**Conclusions:**

Our clinical experience supports the PPITF to be a viable alternative for reconstruction of the skull base in the absence of the nasoseptal flap.

## Introduction

Over the past decade, the role of endoscopic surgery in the management of anterior skull base pathologies has evolved. Expanded endonasal approaches (EEA) provide exposure to skull base and intradural pathology, while reducing the morbidity associated with traditional craniofacial approaches [1-4]. Following tumor extirpation, the resultant cranial base defect requires reconstruction to form a watertight barrier separating the intracranial compartment and sinonasal tract. Failure to achieve adequate separation can lead to complications, including cerebrospinal fluid (CSF) leak, pneumocephalus and meningitis [5,6]. Small fistulae (<1 cm) can be repaired with a high rate of success using a variety of multilayered free grafts [7]. In such cases, long-term prevention of CSF leaks and infections appear to be independent of the material or technique (inlay or overlay) used in the repair [7,8].

Skull base defects resulting from EEAs are more challenging to reconstruct due to their complexity and size. With the increase in frequency of EEAs and variety of pathologies treated, a reconstructive ladder in defect repair is developing. Local vascularized flaps have become the main reconstructive option due to their ease of elevation, low donor site morbidity, low complication rate, and propensity for rapid and complete healing [9,10]. The Hadad-Bassagasteguy flap (HBF), a vascularized pedicled nasoseptal mucoperiosteal flap based on the nasoseptal artery, has significantly decreased the incidence of postoperative CSF leaks [11]. However, the surgeon must consider alternatives to the HBF flap, particularly in situations where it is not available due to tumor involvement of the septum, or in revision cases where a previous septectomy has been performed.

The posterior pedicle inferior turbinate flap (PPITF) has been described as an appropriate alternative [12]. The PPITF is based on the inferior turbinate artery, a branch of the posterolateral nasal artery, which arises from the sphenopalatine artery. This intranasal flap is advantageous compared to regional vascular flaps, including the temporoparietal fascial and pericranial flaps, as it eliminates the morbidity associated with an external incision and minimizes healing time due to rapid mucosalization. Through a case series, we describe our experience with the PPITF.

## Methods

We retrospectively analyzed the demographic, presentation, treatment, follow-up, surgical and outcomes data of two patients who underwent skull base reconstruction with the PPITF at Toronto Western Hospital and Mount Sinai Hospital (Toronto, Ontario, Canada). The Research Ethics Board at each institution approved the study.

### Surgical technique

**Figure 1:**
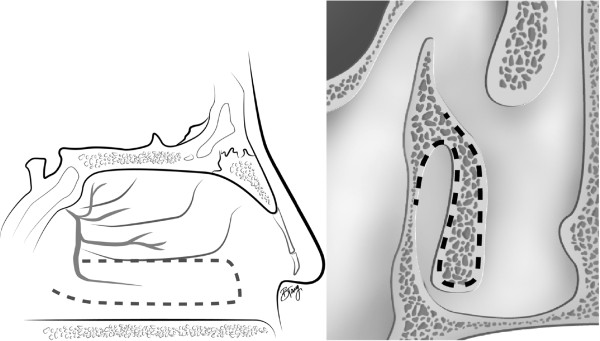
**Planned incisions for the inferior turbinate flap.** Note the sagittal image on the left shows the incision around the pedicle of the inferior turbinate. The coronal image (right) shows the S-shape that is performed at the head of the inferior turbinate.

**Figure 2:**
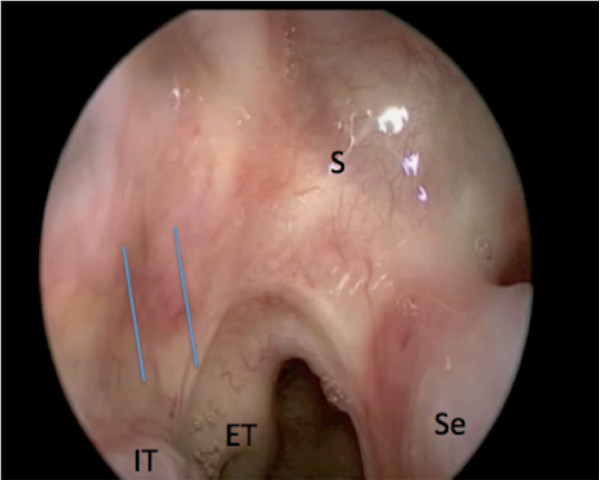
**Endoscopic view of the right posterior nasal cavity.** A posterior septectomy and middle turbinectomy were performed as part of a previous surgery. The parallel lines denote the course of the pedicle of the inferior turbinate. IT: inferior turbinate; ET: eustachian tube; S: sphenoid face; Se: septum.

Elevation of the PPITF usually precedes extirpation of the tumour. Prior to surgery, decongestion of the nasal cavity is achieved with topical adrenaline. The inferior turbinate can be gently medialized to better visualize its medial surface and the mucosa from the inferior meatus, and then subsequently laterally fractured to gain access to the lateral nasal wall. The sphenopalatine foramen is identified superior to the posterior inferior turbinate, posterior to the basal lamellae of the middle turbinate. The planned incisions on a sagittal and coronal model are shown in Figure 1. The pedicle blood supply to the inferior turbinate can sometimes be visualized as pulsating, which aids in incision planning (Figure 2).

**Figure 3:**
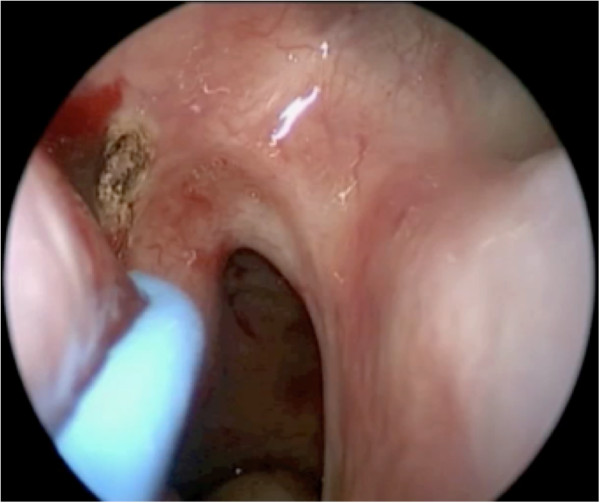
The inferior incision starts posterior to the sphenopalatine foramen.

**Figure 4:**
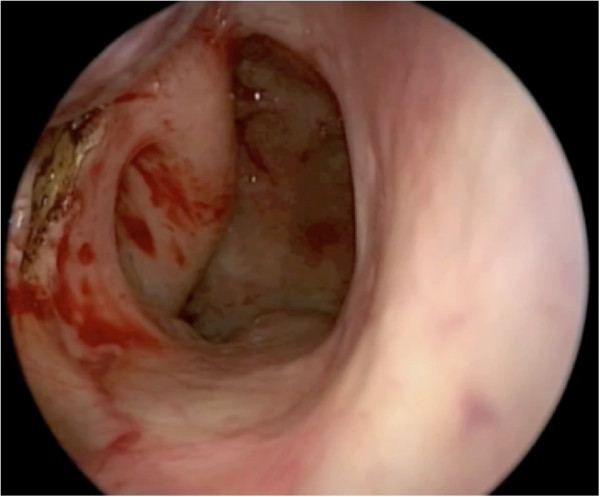
The vertical limb of the posterior incision is seen, anterior to the Eustachian tube.

Next, the submucosa around the pedicle and at the anterior end of the inferior turbinate is infiltrated with 1% lidocaine and epinephrine 1:100,000. Incisions are performed with a needle-tipped monopolar cautery, bent at 45 degrees. The inferior incision starts posterior to the sphenopalatine foramen, and descends vertically anterior to the Eustachian tube, down to the nasal floor (Figures 3 and 4). This is then brought anteriorly to arch up the inferior meatus to the anterior end of the inferior turbinate.

**Figure 5:**
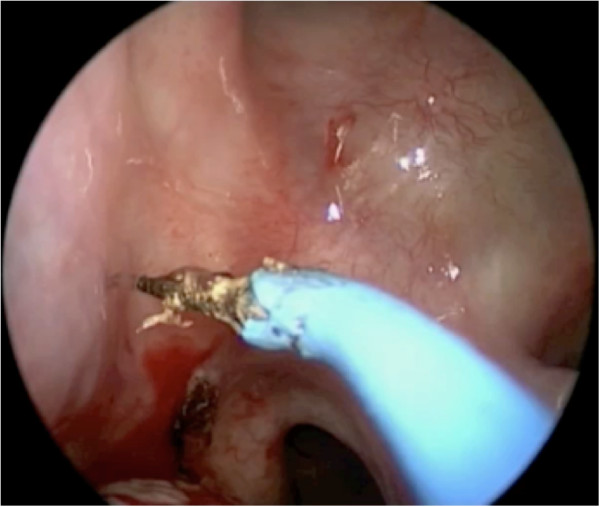
**The superior incision starts anterior to the sphenopalatine foramen.** Note the proximity to the inferior incision.

**Figure 6:**
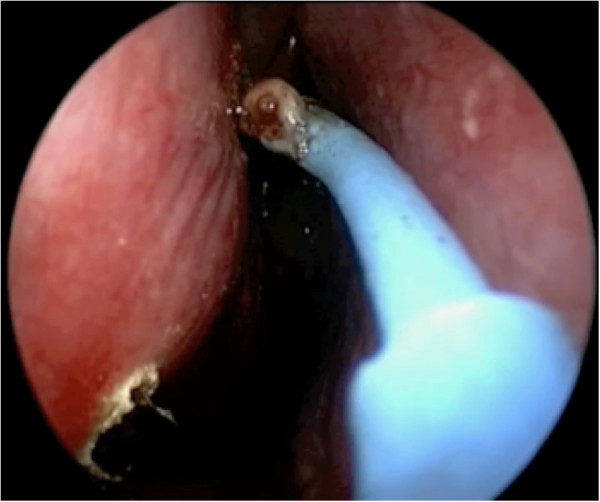
A vertical limb is made anteriorly to connect the two incisions.

The superior incision begins anterior to the sphenopalatine foramen, and continues anteriorly in a horizontal plane over the attachment of the inferior turbinate on the lateral nasal wall (Figure 5). A vertical incision at the head of the inferior turbinate then connects the two incisions (Figure 6). This incision is in an S-shape, starting from the superior incision, sloping around the contour of the head of the inferior turbinate and onto the inferior meatus (Figure 1). Care should be taken to avoid disrupting the valve of Hasner.

**Figure 7:**
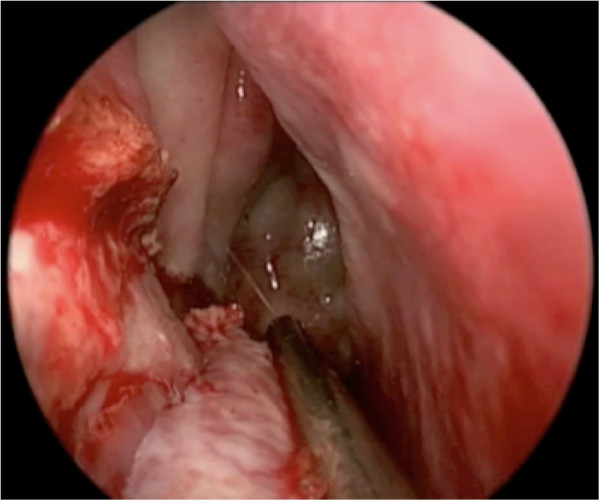
The mucosa is freed off the lateral nasal wall.

**Figure 8:**
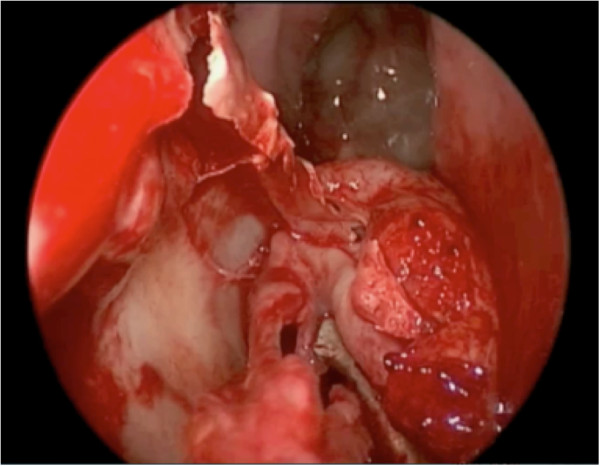
The mucosa is degloved off the bone of the inferior turbinate with a Cottle elevator.

Elevation of the flap is slightly more challenging than for a nasoseptal flap. Careful elevation with a Cottle instrument will help ensure flap viability (Figures 7 and 8). The bone of the inferior turbinate is left in place to re-mucosalize, and therefore minimizing the morbidity of the procedure.

**Figure 9:**
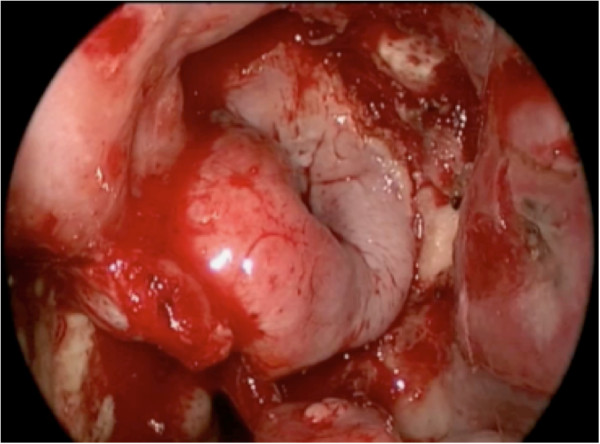
After tumour resection, the flap placed as an overlay.

The flap is then tucked in the nasopharynx, and then brought back up at the end of the case as an overlay on the defect (Figure 9). The flap is smoothed out in its normal rotation so that the mucosal side is facing externally, and the pedicle not kinked. There is a limited arc of rotation compared to a nasoseptal flap, and proper placement of the flap may be more difficult.

A multilayered reconstruction is usually accomplished with a collagen matrix as an underlay (DuraGen®, Integra LifeSciences; Plansboro, NJ, USA) followed by the flap as an overlay. The edges of the flap are covered with Surgicel® (Ethicon; Somerville, NJ, USA) and the whole area is matted with fibrin glue. Saline-soaked Gelfoam® (Pfizer; NY, USA) follows, and a foley catheter is gently inflated and buttressed against the repair. Doyle silastic splints are sutured across the septum.

## Results

### Case reports

Two patients underwent skull base reconstruction using the PPITF.

### Patient 1

A 44 year-old female initially presented with amenorrhea and galactorrhea in 1998, and subsequently underwent a sublabial, trans-sphenoidal approach for resection of a pituitary macroadenoma. The sellar floor was reconstructed with Surgicel**®**, Gelfoam**®** and a bone graft from the septum. Nine years postoperatively, the patient was taken back to the operating room because of recurrent amenorrhea and tumour growth on serial imaging. The surgery at this time included an EEA with posterior septectomy and wide sphenoidectomy. The cranial base defect was reconstructed this time with a middle turbinate mucosal flap.

A recent MRI scan showed residual tumor across the clivus with a nodular component in the prepontine cistern invaginating the anterior surface of the pons. The decision was made to undergo a third operation, and the residual tumor was resected using an EEA via trans-clival and trans-sellar approaches. Due to a previous septectomy, the HBF was unavailable and the defect was repaired with a right PPITF. At 18 days post-operatively, there was no evidence of a CSF leak or infection, and the sellar cavity was healing well.

### Patient 2

A 60 year-old female was referred to our clinic in June 2010 for evaluation of a pituitary adenoma. She initially presented with apoplexy secondary to compression of the optic chiasm by a large pituitary macroadenoma. The patient underwent urgent surgical decompression by EEA via a trans-sphenoidal approach with a right middle turbinectomy, posterior ethmoidectomy and posterior septectomy. A HBF was used to repair her resultant skull base defect at that time. Post-operatively, the patient regained her field of vision in her left eye and her visual acuity remained unchanged. Serial imaging showed a stable residual tumor within the right pituitary fossa, extending into the suprasellar cistern, with minimal compression of the optic chiasm and right proximal optic nerve.

At 17 months post-operatively, she complained of progressive right-sided visual field loss and decline in visual acuity. The residual tumor was resected using an EEA via a trans-sphenoidal approach. The previous nasoseptal flap was removed and a right-sided PPITF was raised to reconstruct the skull base. Excellent coverage was achieved.

In similar revision cases, the senior author (AV) usually attempts to salvage the original nasoseptal flap, as previously described [13]. However, in this case, contraction of the flap (secondary to healing) did not allow for adequate coverage of the resultant defect.

### Follow-up and complications

**Figure 10:**
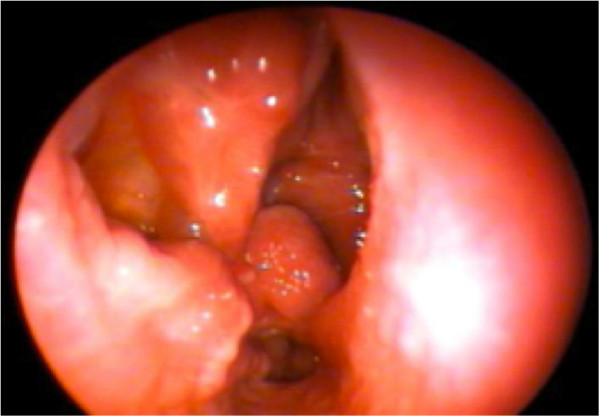
Well-mucosalized nasal cavity in clinic 6 weeks after surgery.

Follow-up revealed that both flaps healed uneventfully and that the PPITFs achieved full coverage of defects. Crusting was common and required frequent debridement until mucosalization was complete. Patients were seen initially at three weeks postoperatively, and then every two weeks until the posterior nasal cavity was clear of granulation tissue. Mucosalization of the lateral nasal wall was observed after 6-8 weeks (Figure 10). At 15 weeks post-operatively, there were no complications from the flap donor site or evidence of CSF leaks.

## Discussion

The basic tenets of skull base reconstruction are to separate the cranial cavity from the sinonasal tract and to protect vital neurovascular structures [11]. A hermetic separation protects against post-operative CSF leaks, ascending bacterial infections, as well as vascular blowouts and pseudoaneurysms secondary to desiccation or infection of major vessels [6]. Although repair of small CSF fistulas are successful in >95% of cases, large dural defects after EEAs pose a complex reconstructive challenge for the endoscopic surgeon. Repair of such defects must take into account additional factors, including the anticipated size, shape and location of the dural defect, intra-arachnoid dissection, previous intranasal or maxillofacial surgeries, previous radiation treatment, and the risk of increased CSF pressure post-operatively [11,14,15]. An ideal reconstruction should be reliable and minimize the complications relating to inadequate closure. The reconstructive ladder for skull base repair is based on the concept of employing the simplest procedure with the highest success rate, regardless of technical complexity [16]. This can be seen as a contrast to the traditional concept, which uses the least complicated and safest procedure available. The ladder currently includes avascular grafts, nasoseptal pedicled flaps, turbinate flaps, and regional flaps [14].

Vascularized flaps provide the most reliable and reproducible reconstruction of large skull base defects [6]. These flaps are also associated with lower postoperative CSF leak rates compared to free tissue grafts [15]. The HBF (i.e. posterior pedicle nasoseptal flap) has become the workhorse of endonasal reconstruction due to its versatility, good arc of rotation and large surface area [6]. The reconstructive surgeon however must consider alternatives when the HBF is not available, for example if there is tumour involvement of the septum or if a previous posterior septectomy was performed (the latter is often the case in revision surgeries). In such scenarios, the PPITF is a suitable alternative [12]. The anatomy and vascular supply of the lateral nasal wall has previously been described [17-20]. Blood supply to the PPITF arises from the inferior turbinate artery, a terminal branch of the posterolateral nasal artery, which in turn is a branch of sphenopalatine artery. Additional contributions are supplied from branches of the facial artery [18].

Historically, pedicled inferior turbinate flaps have been used for the closure of nasal septal perforations and oronasal fistulas [21-23]. Fortes et al. were the first to describe the use of the PPITF for skull base reconstruction. In their series, four patients underwent successful reconstruction with the PPITF after their index operation resulted in posterior cranial fossa defects and nasoseptal flaps were unavailable [12]. Lee et al. recently published a case series of five patients who had skull base defects repaired with the PPITF. With increasing surgical experience and appropriate patient selection, they found the PPITF to be a reliable reconstructive option due to its excellent blood supply [24]. The current series of two patients adds significantly to the aforementioned studies, and it is also the first to describe the use of this complex, technically-advanced procedure at a Canadian institution. We showed that the PPITF was associated with minimal morbidity and postoperative complications. Initial nasal crusting resolved with re-mucosalization of the donor site. Adequate seal and 100% flap survival were achieved in all cases.

There are limitations to the PPITF, including its limited ability to reach the anterior skull base. An anatomic analysis showed that the PPITF could only reach 67% of the length of anterior cranial fossa defects [25]. To address this, an anteriorly based inferior turbinate flap has been shown to cover large portions of the anterior cranial fossa, from the posterior table of frontal sinus to the planum [26]. This flap can be used alone or in combination with the HBF or PPITF with relatively minimal morbidity [25]. Another limitation of the PPITF is that its surface area is not as extensive compared to nasoseptal or extranasal flaps [12,25]. The estimated surface area of the PPITF is 2.4 ± 1.0 cm^2^ 5.4 cm length by 2.2 cm width) [25]. However, additional mucosa extending beyond the lateral nasal wall, including the mucoperiosteum of the inferior lateral wall, inferior meatus, and nasal floor, can be harvested to increase the surface area of the flap [6,27]. It has been suggested that the surface area of these enlarged flaps may be three times greater than the PPITF [6]. In addition, the vascularized mucoperiosteum placed on the centre of the defect and augmented by free grafts may be sufficient to provide a complete, hermetic seal [25]. Finally, in certain situations, bilateral PPITFs can be harvested to ensure complete coverage of the defect.

## Conclusion

As an alternative to the nasoseptal flap, the posterior pedicle inferior turbinate flap (PPITF) is a viable and safe alternative for skull base reconstruction, particularly for posterior cranial fossa defects. Our outcomes were comparable to other case series using the PPITF. Careful preoperative planning is necessary to ensure complete coverage of skull base defects.

## Disclosure

This material has never been published and is not currently under evaluation in any other peer-reviewed publication.
